# Fascin-1 in Cancer Cell Metastasis: Old Target-New Insights

**DOI:** 10.3390/ijms241411253

**Published:** 2023-07-08

**Authors:** Eleonora Sarantelli, Apostolis Mourkakis, Lefteris C. Zacharia, Andreas Stylianou, Vasiliki Gkretsi

**Affiliations:** 1 Biological Sciences Program, Department of Life Sciences, School of Sciences, European University Cyprus, Nicosia 2404, Cyprus; es172241@students.euc.ac.cy; 2 Cancer Metastasis and Adhesion Laboratory, Basic and Translational Cancer Research Center (BTCRC), European University Cyprus, Nicosia 2404, Cyprus; am231067@students.euc.ac.cy; 3 Department of Health Sciences, School of Life and Health Sciences, University of Nicosia, Nicosia 2417, Cyprus; zacharia.l@unic.ac.cy; 4 Cancer Mechanobiology and Applied Biophysics Laboratory, Basic and Translational Cancer Research Center (BTCRC), European University Cyprus, Nicosia 2404, Cyprus; an.stylianou@euc.ac.cy; 5 Biomedical Sciences Program , Department of Life Sciences, School of Sciences, European University Cyprus, Nicosia 2404, Cyprus

**Keywords:** actin cytoskeleton, invasion, migration, invadopodia, mechanobiology

## Abstract

As metastasis is responsible for most cancer-related deaths, understanding the cellular and molecular events that lead to cancer cell migration and invasion will certainly provide insights into novel anti-metastatic therapeutic targets. Fascin-1 is an actin-bundling protein fundamental to all physiological or pathological processes that require cell migration. It is responsible for cross-linking actin microfilaments during the formation of actin-rich cellular structures at the leading edge of migrating cells such as filopodia, lamellipodia and invadopodia. While most epithelial tissues express low levels of Fascin-1, it is dramatically elevated in the majority of cancers and its expression has been associated with more aggressive disease and decreased overall survival. Hence, it has been proposed as a potential anti-cancer target. In the present review, we studied recent literature with regard to Fascin-1 expression in different cancers, its role in altering the mechanical properties of cancer cells, promoting cancer cell migration, invasion and metastasis and the effect of its inhibition, via various pharmacological inhibitors, in eliminating metastasis in vitro and/or in vivo. Recent studies corroborate the notion that Fascin-1 is critically involved in metastasis and prove that it is a valuable anti-metastatic target that is worth investigating further.

## 1. Introduction

### 1.1. Cancer Cell Metastasis

Cancer-related mortality is rarely attributed to the primary tumor but rather to the spreading of cancer cells and the development of metastatic lesions in other sites of the body [1]. Although intensive worldwide research efforts have been made in the field and several anti-cancer targets have been identified and new drugs have been developed, they mostly aim at inhibiting tumor growth. Thus, cancer treatment tends to be largely ineffective with the patient finally dying of overt metastasis. Therefore, understanding the molecular and cellular events in the metastatic process is of vital importance for the identification of metastasis-related biomarkers and the development of novel therapeutic interventions that can target metastasis and inhibit it.

Metastasis is a complex multistep process during which cancer cells initially detach from the primary tumor, migrate and invade the surrounding tissues, intravasate into neighboring blood or lymphatic vessels and travel through the circulation or the lymphatic system, respectively, to other parts of the body where they establish a new tumor [2].

For metastasis to take place, cancer cells need to undergo epithelial to mesenchymal transition (EMT) whereby epithelial cells are accumulating mutations and are slowly acquiring mesenchymal cell-like characteristics which include but are not limited to expression of mesenchymal-related genes, change in shape and increased migratory and invasive capacity [3]. During this process, actin cytoskeleton is being reorganized to form actin-based cellular protrusions that will facilitate the cell’s migration and invasion through adjacent tissues. These structures, namely filopodia, lamellipodia and invadopodia start forming at the invasive front and are rich in actin microfilaments that are tightly bound together [4].

### 1.2. Actin-Based Structures in Metastasis: Filopodia, Lamellipodia and Invadopodia

Filopodia are slender, finger-like projections that extend from the cell surface and consist of actin-filament bundles that are arranged in a parallel manner. They are considered to play a crucial role in cell migration, cell adhesion, sensing the environment, establishing cell–cell contacts and cell–cell communication and guiding cells during normal development.

Lamellipodia are broad, sheet-like extensions of the cell membrane rich in branched actin networks that are typically found at the leading edge of migrating cells. They are involved in cell motility by generating pushing forces that propel the cell forward thus being responsible for the protrusion of the cell membrane during migration, facilitating the cell’s exploration of the extracellular environment [5].

Finally, invadopodia are also actin-rich, specialized membrane protrusions that also possess a matrix-degrading function being enriched in various proteins involved in matrix degradation, including matrix metalloproteinases (MMPs) [6]. They are typically found in invasive cells, such as cancer cells, that are capable of penetrating through surrounding tissues.

As all these structures are based on the organization of actin cytoskeleton, they are influenced by signaling pathways affecting actin-organization such as the Rho GTPases whose dysregulation has also been implicated in cancer [7,8]. Apart from Rho GTPases, with main members being RhoA, Rac-1 and Cdc42, there are a number of proteins involved in the regulation of actin dynamics that may ultimately affect the formation of filopodia, lamellipodia and invadopodia as well as their number and distribution finally altering cellular function.

### 1.3. Fascin-1 in Normal Cells and Tissues

Fascin-1 is 55 KDa protein, member of the Fascin family of actin-binding proteins, coded by a gene located on chromosome 7p22 [9], which has been found to be responsible for cross-linking F-actin microfilaments into tight, parallel bundles that are required for the formation of the actin-rich migratory structures of filopodia, lamellipodia and invadopodia [4]. Interestingly and in addition to actin binding, Fascin-1 is responsible for other functions, beyond reorganization of cellular cytoskeletal structures, including modulating the activity of actin-binding proteins that regulate microtubule dynamics and mediating mechano-transduction through the linker of nucleoskeleton and cytoskeleton (LINC) complex [10].

**Figure 1:**
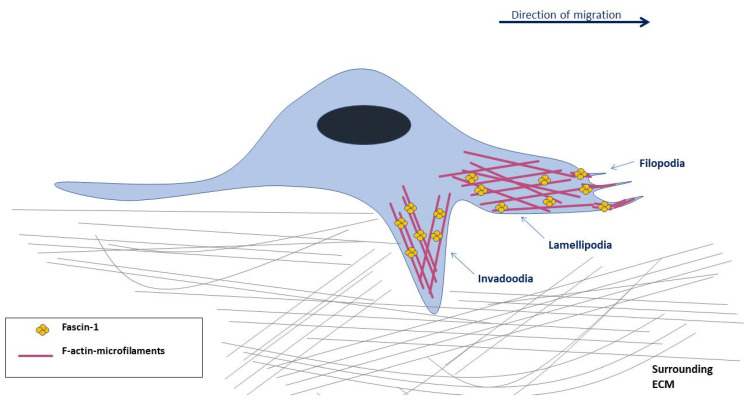
Schematic representation of a cancer cell forming actin-rich invadopodia, lamellipodia and filopodia in attempt to invade through surrounding extracellular matrix (ECM) and migrate towards a specific direction. Protein Fascin-1 is shown to mediate actin filament bundling within the structures of filopodia, lammelipodia and invadopodia.

Fascin-1 is a globular protein consisting of four β-trefoil domains organized in such a way that one molecule of Fascin-1 is sufficient to cross-link two actin microfilaments as there are more than one actin-binding sites available per molecule (Figure 1).

Moreover, these actin-binding sites are highly coordinated with each other to such an extent that compromising one impairs the binding capacity of the others [10].

In humans, the expression of Fascin-1 is higher during development and is noticeably absent from normal epithelial tissues [11], although it is highly expressed in neurons, the glomerulus, the adrenal glands, the basal layer of the skin, immune cells and especially dendritic cells, which are more motile [10].

### 1.4. Fascin-1 and the Mechanical Properties of the Cell

Considering the fact that Fascin-1 regulates actin-cytoskeleton organization, it is only plausible to assume that it also plays an important role in regulating the mechanical properties of the cell. Indeed, in a recent study by Tanaka et al. using super-resolution microscopy to visualize the actin meshwork of lamellipodia in live growth cones showed that not only Fascin-1 colocalizes with the actin meshwork in lamellipodia but it also contributes to the elasticity of the growth cone [12]. Thus, dissociation of actin filaments from Fascin-1 molecules leads to increased mechanical flexibility that plays a crucial role in enabling growth cones to penetrate various tissues, thus enabling cancer cell invasion and metastasis.

In another study, Park et al. combined experimental and computational techniques to study how solution crowding influences the organization and mechanical properties of Fascin-1-induced bundles [13]. Since it is known that crosslinked bundle formation occurs in crowded cytoplasm, they investigated the impact of crowding on the bundling activities of Fascin-1 crosslinking showing that crowding can alter the stiffness and helical twist of filaments, leading to distinct organization and mechanics of the crosslinked bundles. Specifically, in the case of Fascin-1, solution crowding results in densely packed bundles and disrupts the binding of Fascin-1 to actin filaments. Also, external factors can influence cells’ mechanical properties. The mechanical stresses exerted on cytoskeletal networks can impact the binding characteristics of cross-links, consequently affecting cytoskeletal reorganization in response to force and altering force transmission within the cell. For instance, when actin bundles are cross-linked by Fascin-1, the forced dissociation of cross-links can transpire at varying rates under different levels of applied stress [14]. Consequently, this phenomenon can induce a softening effect on the system through mechanical means, which has also been associated with a more aggressive cancer phenotype [15].

While these findings suggest a role for Fascin-1 in controlling the mechanical properties of cells with obvious implications in cancer progression, it is important to note that the exact mechanisms by which Fascin-1 affects these processes are still vague.

### 1.5. Fascin-1 in Cancer Cells and Tissues

In contrast to normal tissues, increased Fascin-1 expression has been found in many cancer tissues such as colorectal cancer (CRC), lung, liver and prostate cancer [16] and has been shown to correlate with aggressive disease, poor prognosis and high mortality risk as well as metastasis [17,18].

### 1.6. Colorectal Cancer (CRC)

In CRC, in particular, a study by Piskor et al. assessed Fascin-1 expression in 51 CRC patient samples using immunohistochemistry. Out of the 51 CRC samples, 17 showed lymph node metastasis and 10 had micrometastases in the liver and lungs. Interestingly, while Fascin-1 was not detected in normal colon mucosa, it was very prominent in CRC samples, indicating that increased Fascin-1 expression is more common in the unfavorable clinical type of adenocarcinoma without mucosal component. However, statistical analysis did not demonstrate statistically significant association between Fascin-1 expression and age, sex, tumor localization, histopathological grade, local and distant metastasis, cancer depth, or intestinal cellular and inflammatory infiltration [19] (Table 1). In another study by Tampakis A. et al., Fascin-1 expression was evaluated in 111 CRC samples, and it was found significantly higher in clinical stage III and IV compared to stage I and II. Patients with vascular invasion and metastatic disease also showed significantly higher Fascin-1 expression, suggesting that Fascin-1 could be a potential biomarker to identify patients prone to disease recurrence, either manifesting as local recurrence or metastasis [20]. The expression levels of Fascin-1 were also examined in 360 CRC patients who had undergone primary surgical treatment. In this study, patients with positive Fascin-1 expression presented worse remission-free survival (RFS) and overall survival (OS) [21]. Thus, increased Fascin-1 expression is shown to be correlated with a more aggressive phenotype and reduced OS in CRC samples, suggesting that Fascin-1 should be evaluated as a potential CRC biomarker.

### 1.7. Lung Cancer

Lung cancer can be found in two main forms: non-small cell lung cancer (NSCLC), which is the most common type, and small cell lung cancer (SCLC). According to a study by Ling et al., high Fascin-1 expression in 128 NSCLC tissue samples was found to be significantly correlated to lymph node metastasis and reduced OS [22]. Similarly, Zhang et al. evaluated 61 NSCLC samples and found that Fascin-1 was expressed at higher levels (70.5%) in cancer compared to normal adjacent tissues (13.1%) and was also associated with tumor diameter and mediastinal lymph node metastasis [23]. Along the same line, Zhao et al. used immunohistochemistry to study the expression of Fascin-1 in 84 paraffin-embedded lung cancer samples, 37 of which were classified as stage I, 32 cases as stage II and 15 cases as stage III based on TNM staging. The results showed that although there was no statistical difference in groups of patients with different sexes, there was a corresponding increase in Fascin-1 expression level as age and clinical staging advanced. Furthermore, the intensity of Fascin-1 protein expression in lung cancer tissues was negatively associated with patients’ OS [24].

The role of Fascin-1 in promoting lung cancer aggressiveness was also shown in vitro in A549 and H520 NSCLC cell lines. *Fascin-1* gene silencing by short-hairpin RNA (shRNA) led to inhibition of the mitogen-activated protein kinase (MAPK pathway) and concomitant inhibition of proliferation, migration and invasion of lung cancer cells, indicating that Fascin-1 by itself promotes an aggressive cancer phenotype [25] (Table 2).

### 1.8. Breast Cancer

In breast cancer, Fascin-1 is responsible for invadopodia formation, migration and invasion of cancer cells, and it has been shown to be a direct target of the canonical Transforming Growth Factor β (TGFβ)-Smad4 signaling pathway which, in breast, has been found to be under the control of GATA-3 transcription factor, a factor involved in breast morphogenesis [26]. With regard to breast cancer tissue samples, Min et al. used immunohistochemistry to evaluate Fascin-1 expression in a microarray consisting of 194 samples from patients diagnosed with invasive breast carcinoma (IDC). The findings revealed a significant association between Fascin-1 expression and several clinicopathological parameters, including high histological grade, tumor necrosis, high expression of p53 and Ki-67 and negative status of estrogen and progesterone receptors. Importantly, a significant negative correlation was found between Fascin-1 expression, RFS and OS, indicating that Fascin-1 is critical for predicting aggressive tumor behavior, particularly in patients with advanced-stage breast cancer [27]. In another study, Wang et al. examined immunohistochemically a total of 457 breast cancer samples, 82 of which were triple negative breast cancer (TNBC) samples, which are considered to be a highly aggressive breast cancer subtype giving rise to higher metastasis rates. Positive expression of Fascin-1 was found in 31.5% of all breast cancer cases (144/457), with 16.8% (77) being strongly positive. Notably, positive expression of Fascin-1 was significantly higher in TNBC cases than in the other molecular subtypes, suggesting that Fascin-1 expression is correlated to increased metastasis. The findings also suggested that the strong positive expression of Fascin-1 could serve as a new diagnostic marker for TNBC samples [28]. Notably, total genomic DNA was isolated from the blood of 316 breast cancer samples and 222 healthy controls for the detection of six (6) Fascin-1 single nucleotide polymorphisms (SNPs) in relation to breast cancer susceptibility and clinicopathological characteristics. Breast cancer cases were categorized according to the expression of estrogen receptor (ER), progesterone receptor (PR) and human epidermal growth factor receptor 2 (HER2). Results showed that breast cancer patients with the Fascin-1 rs3801004 polymorphism had a higher risk of stage III/IV disease progression and lymph node metastasis. Similarly, the Fascin-1 rs2966447 polymorphism was associated with a higher risk of pathologic disease while rs2966447 polymorphism was associated with a higher risk of developing stage III/IV disease and lymph node metastasis in the Luminal A subgroup, as well as a higher risk of pathologic grade disease in the Luminal B subgroup. Thus, it was shown that genetic variations in the *Fascin-1* gene may predict early-stage breast cancer [29].

Finally, in a study involving 127 breast cancer samples and equal number of benign breast tissues from the same patients, although no correlation was observed between Fascin-1 expression and the stage of the tumor or the presence of lymph node metastasis, a significant association was revealed between high Fascin-1 expression, TNBC cases, high-grade tumors, high expression of the cell proliferation marker Ki-67 and expression of Fascin-1 in the endothelial cells of tumor vessels. Moreover, patients with high Fascin-1 expression had significantly lower rates of RFS [30].

### 1.9. Prostate Cancer

In prostate cancer, Abosarie and Ibrahim assessed the expression of Fascin-1 in 80 prostate cancer samples using immunohistochemistry. Their results showed positive expression of Fascin-1 in 90% of prostate cancer cases while a statistically proven relationship was also found between Fascin-1 expression and prostate-specific antigen (PSA) level, Gleason score as well as lymphovascular and perineal invasion. In fact, Fascin-1 expression increased with aggressiveness of prostate cancer, suggesting that it can be used as a prognostic marker to predict poor outcome in patients with prostate cancer [31].

Similarly, in another recent study, Fascin-1 expression was evaluated at the mRNA and protein level in 20 cases of prostate cancer tissue and 20 cases of histologically normal adjacent tissue, and it was found that Fascin-1 expression was significantly elevated in the prostate cancer group compared to the normal adjacent tissue group. Moreover, evaluation of Fascin-1 expression in human normal prostate epithelial cells WPMY-1 and human prostate cancer cells PC-3 and DU145 verified this result as Fascin-1 expression was significantly lower in the normal prostate WPMY-1 cells compared to the prostate cancer cells while further in vitro experimentation showed that it promoted prostate cancer cell invasion, migration and EMT [32].

However, immunohistochemical analysis of Fascin-1 protein expression on a tissue microarray that contained a total of 211 low, intermediate and high Gleason risk prostate cancer samples showed that although Fascin-1 expression tended to be elevated more frequently in tumor cells proportional to high Gleason score versus low Gleason score tumors, this was not statistically significant. More specifically, only 8% of tumors in the tissue microarray contained >10% Fascin-1 positive carcinomas, which were usually detected in variable, focal patches, which further increased the complexity of evaluation and scoring [33].

Finally, in another line of investigation, Tataru et al. collected blood samples from 62 prostate cancer patients and 61 age-matched control male patients without abnormal digital rectal examination or known malignant disease and assessed the serological values of Fascin-1 by quantitative sandwich ELISA but did not observe any significant difference in expression [34]. This finding may initially seem contradictory to the pro-metastatic role of Fascin-1 seen in all other cancer types. However, taking into account the fact that the evaluation in this study was performed in blood samples and not in the actual cancer tissue, this immediately changes the parameters of the comparison between the studies. Hence, this discrepancy may simply indicate that Fascin-1 expression should not be evaluated in the blood serum but rather in the actual affected tissue.

### 1.10. Osteosarcoma

Fascin-1 was also studied in osteosarcoma [35]. Specifically, Fascin-1 expression was evaluated by immunohistochemistry on a tissue microarray constructed from osteosarcoma and normal bone tissue specimens from 67 patients in Switzerland. Kaplan–Meier survival analysis of Fascin-1 expression in osteosarcoma samples revealed a direct correlation between Fascin-1 expression and poor patient OS. Moreover, in this study, Arlt et al. continued to verify their results in vitro using SaOS-2 and 143B osteosarcoma cells. Overexpression of Fascin-1 in OS cells promoted their migratory capacity as well as the activity of the MMP-9, which is critically involved in the metastatic process. Fascin-1 silencing was shown to lead to reduced formation of filopodia in vitro, while overexpression had no effect on the cell shape. Further, in vivo experiments using two different xenograft mouse models and orthotopic intratibial transplantation of the two different osteosarcoma cell lines showed that overexpression of Fascin-1 also enhanced malignant phenotype compared to control and promoted tumor growth and lung metastasis in vivo [35]. This study used in vitro and in vivo experiments in combination with findings from clinical samples and proved that Fascin-1 indeed has potential as a novel prognostic biomarker in osteosarcoma and could be a potent therapeutic target to halt metastasis and improve patient OS.

In another study, the localization and organization of organelles and actin regulatory proteins was assessed in human U2OS osteosarcoma cells and A375M melanoma cells undergoing migration under the mode of “leader bleb-based migration”, which is typically characterized by long bleb towards the direction of the movement that is separated from the rest of the cell body by a distinct contractile “neck”. In addition to its anticipated localization in the cell body, Fascin-1 exhibited a distinct and prominent localization in the leader bleb. Further, Fascin-1 overexpression increased migration speed while *Fascin-1* siRNA-mediated silencing impaired cell migration and showed Fascin-1 as a critical player in the process [36].

### 1.11. Stomach Cancer

In stomach cancer, Tu et al. used 204 gastric cancer samples and assessed Fascin-1 protein expression by immunohistochemistry and immunoblotting. The rate of positive Fascin-1 expression was 45.1% (92/204) in gastric cancer tissues, which was much higher than that of adjacent cancer tissues with 27.5% (56/204). Moreover, protein expression was closely associated with tumor size, while it was not found to be associated with age, sex, depth of invasion, clinical staging, differentiation, lymph node metastasis, distant metastasis and vascular invasion. This indicated that Fascin-1 is an important factor in tumor growth but not necessarily metastasis of stomach cancer cells [37]. In another study, normal human gastric mucosal GES-1 cells and various human gastric cancer cell lines, including poorly differentiated MKN45 cells, moderately differentiated SGC-7901 cells and highly differentiated MKN28 cells, were utilized along with gastric cancer samples and corresponding tumor-adjacent tissues from patients. *Fascin-1* mRNA and protein expression in gastric cancer samples was significantly higher than in adjacent normal tissue while its expression varied among gastric cancer cell lines being increased as the cell differentiation decreased. In vitro experiments involving *Fascin-1* shRNA-mediated silencing resulted in reduced cell migration with concomitant decrease in MMP2, MMP9, vimentin and other mesenchymal makers such as fibronectin and N-cadherin as well as an increase in E-cadherin. These findings indicated that Fascin-1 is closely related to the gastric cancer EMT process and thus metastasis. Finally, in vitro findings were corroborated by in vivo experiments in nude mice injected with control cells or cells lacking *Fascin-1. Fascin-1* silencing had a dramatic effect in the formation of tumors as it inhibited gastric tumor formation in the xenograft model [38].

### 1.12. Hypopharyngeal Squamous Cell Carcinoma (HSCC)

Bu et al. used cultures from the FaDu, hypopharyngeal cancer cell line (HSCC) as well as tumor and adjacent normal tissues collected from 96 patients with HSCC. In vitro experiments showed that Fascin-1 leads to increased formation of filopodia and increased migratory and invasive capacity of FaDu cells while it was also shown to be upregulated by Hypoxia Inducible Factor 1α (HIF1α). Moreover, immunohistochemical analysis showed that Fascin-1 was overexpressed in HSCC tissues and significantly associated with lymph node metastasis [39], suggesting that Fascin-1 may very well be an anti-metastatic therapeutic target in HSCC.

### 1.13. Pancreatic Duct Adenocarcinoma (PDAC)

Another type of cancer in which Fascin-1 protein expression was examined is pancreatic cancer. The study by Misiura et al. included 70 samples from patients who underwent surgery for different pancreatic diseases, 38 of which involved cases of ductal adenocarcinoma. Fascin-1 expression was evaluated by immunohistochemistry, and it was found prominent in the cytoplasm of pancreatic ductal epithelial cells. Further analysis showed that positive Fascin-1 expression in pancreatic intraepithelial neoplasia (PanIN), a precursor lesion of pancreatic ductal adenocarcinoma (PDAC), significantly correlated with the age of the patients being elevated in patients over 60 years of age, as well as the presence and grade of PanIN, indicating that Fascin-1 may play a role in the transformation of PanIN to PDAC [40]. In another study on PDAC, Li et al. investigated Fascin-1 expression in 122 primary human PDAC tissues and further validated their findings using a mouse model. Specifically, they used KRas(G12D) p53(R172H)Pdx1-Cre (KPC) mice to investigate the effects of protein deficiency on the development of PanIN, PDAC and metastasis. Immunohistochemical analysis of PDAC samples from human patients showed that Fascin-1 was absent from the normal pore but was quite prominent in the PDAC cytoplasm. In fact, although Fascin-1 expression levels did not correlate with tumor stage, perineal invasion and lymphatic invasion, 95% of human PDAC expressed Fascin-1 and this expression was highly associated with decreased OS, high tumor grade and vascular invasion. Data from the in vivo experiments corroborated the notion that Fascin-1 is critically involved in PDAC metastasis as they showed that while pancreatic ducts from control mice and early-stage PanINs from KPC mice were negative for Fascin-1 expression, 6% of PanIN and 100% of PDAC expressed the protein. Moreover, Fascin KPC-Cre-deficient mice had longer survival time and delayed PDAC onset than KPC mice, which suggests that Fascin-1 itself promotes formation of PDAC from PanIN without affecting PDAC invasion into the intestine or peritoneum in mice. Finally, the in vitro experiments showed that Fascin-1 was concentrated in filopodia and was proved to be essential for their assembly while it also promoted filopodia formation and cell invasion [41].

### 1.14. Glioma

An in vitro study by Hoa et al. investigated the effect of *Fascin-1* silencing in glioma cells. Glioma is the most common malignant tumor of the brain characterized by high rates of morbidity, mortality and recurrence. In this study, *Fascin-1* was silenced by means of either siRNA or shRNA in U251glioma cells, and both silencing approaches resulted in cells losing their filopodia and acquiring a more rounded squamous appearance as shown using both fluorescence and atomic force microscopy (AFM). Moreover, *Fascin-1* knockdown cells had a slower in vitro growth rate and became less invasive as demonstrated by their inhibited ability to invade through pores in response to interleukin-6 (IL-6) or insulin-increasing factor-1 (IGF-1). Fascin-1 silencing was also shown to improve the cytolytic activity of lymphocytes, while simultaneously slowing glioma growth and inhibiting their ability to migrate in vitro. Thus, it was concluded that strategies that inhibit Fascin-1 expression can enhance immunotherapy against various forms of glioma by three different mechanisms: reduced growth rates, reduced invasiveness and improved cytolytic potential of T cells [42]. Another study by Zhang et al also dealt with glioma but involved human patients’ samples. In this study, glioma samples and normal adjacent brain tissues were collected from 120 patients who underwent surgery. Although high Fascin-1 expression was not found to be associated with other clinicopathological variables, such as age, sex, tumor size and extent of resection, high expression of Fascin-1 was correlated with poor prognosis for glioma patients [43].

### 1.15. Ovarian Cancer

As ovarian cancer cells usually metastasize to surrounding organs through the mesothelial cell layer covering the organs found in the peritoneal cavity, Yoshihara et al. investigated cancer cell trans-mesothelial migration in ovarian cancer cell lines (ES-2, SK-OV3 and NOE cells). Specifically, they developed an in vitro trans-mesothelial migration assay using a layer of primary peritoneal mesothelial cells on top of which ovarian cancer cells were added. Using this method, they showed that *Fascin-1* silencing in ES-2 cells significantly reduced trans-mesothelial migration of cancer cells, whereas overexpression of GFP-Fascin-1 in SK-OV-3 cells had the opposite effect [44].

Another study that focused on ovarian cancer also showed how inhibition of Fascin-1 affects ovarian cancer cell metastasis. In this study, Fascin-1 expression was first assessed in human ovarian cancer cell lines (HeyA8, Ovcar5, Tyk-nu, Snu119 and Kuramochi) and 201 ovarian cancer tissues on a tissue microarray using immunofluorescence and immunohistochemistry, respectively. Results demonstrated that Fascin-1 was highly expressed in the cancer compartment of the tumors in 125 out of 201 samples and in almost every case in the stromal cell compartment (94.0%). Fascin-1 expression was correlated with pathology sub-type and age at diagnosis, but no significant correlation was found between Fascin-1 levels and overall survival or progression-free survival in patients with serous ovarian cancer. However, Fascin-1 expression was associated with worse overall survival but not progression-free survival in non-serous ovarian cancer. No difference was also detected between primary and metastatic tumors as well. In vitro experiments where Fascin-1 was either silenced by siRNA or shRNA or inhibited by a pharmacological inhibitor (compound G2) showed decreased cancer cell migration and impaired Cdc42 and Rac1 activity. Moreover, in vivo experiments showed reduced ovarian cancer metastasis formation in mice. The study revealed the therapeutic potential of pharmacological inhibition of Fascin-1 [45].

Table 1 below presents a summary of the studies employing immunohistochemistry to assess Fascin-1 expression in tissue samples from patients with various cancer types while.

Table 2 below summarizes recent in vitro and/or in vivo studies on the role of Fascin-1 in cancer cell metastasis.

**Table 1:** Summary of findings from studies involving immunohistochemical analysis of Fascin-1 expression in tissue samples from patients with various cancer types.

Cancer Type	Reference	Samples	High Expression Levels Correlated with More Invasive Phenotype	High Expression Levels Associated with Reduced Overall Survival (OS)
Colorectal cancer (CRC)	[19]	51 CRC patient samples	No	N/A
	[20]	111 CRC patient samples	Yes	Yes
	[21]	360 CRC patient samples	Yes	Yes
Lung cancer	[24]	84 specimens of lung cancer tissues and paracarcinoma tissues	Yes	Yes
	[22]	NSCLC tumour tissues from 128 patients	Yes	Yes
	[23]	61 NSCLC patients (46 cases of stage I/II cancer and 15 with stage III cancer)	Yes	N/A
Breast Cancer	[27]	194 tissue samples from patients with IDC of the breast	Yes	Yes
	[28]	457 tissue samples from patients with breast cancer	Yes	Yes
	[30]	127 breast cancer samples and equal number of benign breast tissues from the same patients	Yes	Yes
Prostate Cancer	[31]	80 tissue samples from patients with prostatic carcinoma	Yes	N/A
	[33]	Tissue microarray of prostate tumor samples containing 211 prostate samples of low, intermediate and high Gleason risk score	No	N/A
	[32]	20 cases of prostate cancer tissue and 20 of normal adjacent tissue	Yes	N/A
	[34]	Blood samples from 62 prostate cancer patients and 61 age-matched control male patients—assessment of serological values of Fascin-1	No	N/A
Osteosarcoma	[35]	Osteosarcoma and normal bone tissue specimens from 67 patients	Yes	Yes
Gastric cancer	[37]	204 Gastric cancer tissues and paired adjacent normal tissues	Yes	Yes
	[38]	50 pair gastric tumor tissue and matched adjacent tissues	Yes	N/A
Hypopharyngeal squamous cell carcinoma (HSCC)	[39]	Tissues and adjacent normal tissues from 96 patients with HSCC	Yes	No
Pancreatic adenocarcinoma (PDAC)	[41]	122 primary human resected PDAC tissues	Yes	Yes
	[40]	70 patients with PDAC	Yes	N/A
Glioma	[43]	Glioma and normal adjacent brain tissue samples from 120 patients	Yes	Yes
Ovarian Cancer	[45]	Tumor microarray made of 201 ovarian cancer tissue samples	No	Only in non-serous ovarian cancer

N/A: non-appliccable, corresonds to studies that do not provide information on OS.

### 1.16. Fascin-1 Inhibitors

Taking into account all the above, Fascin-1 inhibitors aiming to block the Fascin–actin interaction can be promising anti-metastatic agents. Despite the fact that targeting protein–protein interactions can be challenging, in the last few years, a number of inhibitors have been synthesized, and their anti-metastatic potential has been proved in many in vitro and in vivo studies. The non-cytotoxic mechanism of action of such inhibitors make them especially promising as they can be coupled with other chemotherapeutic drugs to increase overall efficacy. Importantly, one such inhibitor has progressed to Phase I clinical trial with positive results, and a Phase I/ Phase II study is under way (https://clinicaltrials.gov/ct2/show/NCT03199586, https://clinicaltrials.gov/ct2/show/NCT05023486, accessed on 7 June 2023).

**Figure 2:**
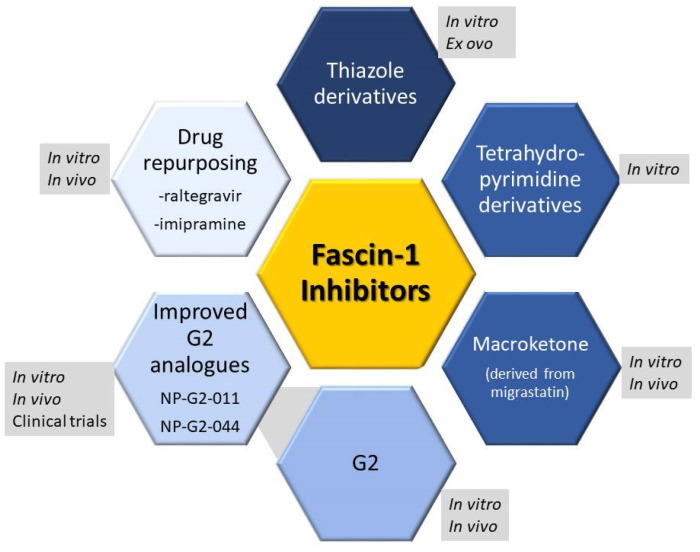
Diagram depicting available Fascin-1 inhibitors and the experimental approach in which they have been tested (in vitro, in vivo, ex ovo or in clinical trials).

Fascin-1 inhibitors comprise a variety of chemical structures all aiming at blocking the Fascin–actin interaction by binding to specific sites on Fascin-1 that facilitate this interaction. An overview of the available Fascin-1 inhibitors is provided in Figure 2.

**Table 2:** Studies on the role of Fascin-1 in cancer cell metastasis using in vitro and/or in vivo experimentation.

Reference	Samples	Methodology	Major Findings
[25]	A549 and H520 NSCLC cell lines	-shRNA mediated *Fascin-1* silencing -MTT survival assay -Migration and invasion assays	*Fascin-1* silencing inhibits MAPK pathway, proliferation, migration and invasion
[29]	318 blood samples from patients with breast cancer and 222 control from healthy participants.	-TaqMan SNP genotyping assay of six Fascin-1 SNPs -Bioinformatics analysis	Association of Fascin-1 SNPs with breast cancer
[26]	MDA-MB-231, MDA-MB-468, MCF-7 and BT474 breast cancer cells	-Invadopodia formation assay -Luciferase assay -Chromatin immunoprecipitation -Real-time PCR -Cell migration and invasion assay	Fascin-1 is responsible for invadopodia formation, migration and invasion of breast cancer cells, and it is a target of (TGFβ)-Smad4 signaling pathway which, in breast, has been found to be under the control of GATA-3.
[35]	-Human SaOS-2 and 143B osteosarcoma cells -Severe combined immuno-deficiency mice	-Immunofluorescence -Wound-healing migration assay -Zymography -Immunoblotting -Human osteosarcoma xenograft mouse model	In vitro: -overexpression of *Fascin*-1 promoted migration and invasion and increased MMP-9 levels -*Fascin-1* silencing reduced formation of filopodia In vivo: overexpression of Fascin-1 in xenograft mouse models enhanced malignant phenotype and promoted tumor growth and lung metastasis
[36]	Human U2OS osteosarcoma cells and A375M melanoma cells	-AFM -Immunocytochemistry -Cell migration speed -siRNA silencing and re-expression	In vitro: Fascin-1 overexpression increased migration speed while *Fascin-1* siRNA-mediated silencing impaired cell migration
[38]	-Normal human gastric mucosal GES-1 cells -Human gastric cancer cell lines MKN45, SGC-7901 and MKN28	-siRNA silencing -Real-time PCR -Immunoblotting -Cell migration assay -Nude mice	In vitro: *Fascin-1* silencing reduced cell migration, MMP-2 and MMP-9 expression and expression of mesenchymal markers In vivo: nude mice injected with cells lacking Fascin-1 had reduced gastric tumor formations
[39]	Human FaDu HSCC cell line	-siRNA silencing -Immunoblotting -Wound healing assay -Transwell assay	In vitro: -Fascin-1 increased formation of filopodia and increased migratory and invasive capacity -Fascin-2 was upregulated by HIF1α
[41]	-PDAC cell lines that were generated from primary pancreatic tumors -KRas(G12D) p53(R172H)Pdx1-Cre (KPC) mice	-Immunoblotting -Real-Time PCR -siRNA Treatment -Live Cell Imaging -Cell Growth Assay	In vitro: Fascin-1 was found in filopodia, and it promoted filopodia formation and cell invasion In vivo: -Fascin KPC-Cre deficient mice had longer survival time, and delayed PDAC onset than KPC mice -Fascin-1 promotes formation of PDAC from PanIN
[42]	Human U251 glioma cells	-siRNA or shRNA *Fascin-1* silencing -Real-Time PCR -Flow cytometry -AFM -Migration assay	In vitro: *Fascin-1* silencing resulted in inhibition of filopodia formation, slower growth rate, reduced invasion, improved cytolytic activity of lymphocytes and inhibited migration
[44]	-ES-2, SK-OV-3 and NOE cell lines -mice	-siRNA mediated *Fascin-1* silencing -Immunoblotting -Trans-mesothelial migration assay -Intraperitoneal injection of ovarian cancer cells in mice	In vitro: *Fascin-1* silencing in ES-2 cells reduced trans-mesothelial migration of cancer cells, whereas overexpression of GFP-Fascin-1 in SK-OV-3 cells had the opposite effect
[45]	-ovarian cancer cell lines HeyA8, Ovcar5, Tyk-nu, Snu119, and Kuramochi	-siRNA or shRNA mediated *Fascin-1* silencing -Pharmacological inhibition of Fascin-1 (compound G2) -Immunofluorescence -Transwell migration assay -Ex vivo colonization assay -In vivo metastasis assay	In vitro: Fascin-1 silencing or pharmacological inhibition decreased cancer cell migration and impaired Cdc42 and Rac1 activity In vivo: reduced ovarian cancer metastasis formation in mice.

Thiazole derivative Fascin-1 inhibitors inhibited migration and invasion in vitro in MDA-MB-231 breast cancer cells as well as angiogenesis in chick embryo ex ovo in chorioallantoic membrane (CAM) assays [46]. Similarly, the tetrahydropyrimidine derivatives inhibited 4T1 breast cancer cell migration [47]. In vivo anti-metastatic activity of these inhibitors has not yet been reported.

Another inhibitor, Macroketone, derived from the natural product Migrastatin, blocks the Fascin–actin-binding sites and was found to inhibit metastatic tumor cell migration and invasion in vitro and metastasis in vivo in mice using the breast cancer cells MDA-MB-231 and murine 4T1 cells [48].

Importantly, compound N-(1-(4-(trifluoromethyl)benzyl)-1H-indazol-3-yl) furan-2-carboxamide, termed G2, selected from screening 165,000 compounds has been shown to inhibit actin-bundling activity of Fascin-1 at a half-maximal inhibitory concentration (IC50) of 5–8 μM. Accordingly, it was also shown to block filopodial formation, tumor cell migration and invasion in vitro using breast cancer cells MDA-MB-231 and murine 4T1 cells and metastasis to lung of mammary carcinoma cells 4T1 [11]. G2 was also shown to decrease migration of cancer and stromal cells in vitro, and in vivo reduced ovarian cancer cell (HeyA8) metastasis to omentum in mice [45]. The efficacy of G2 in inhibiting migration and invasion was also proved in CRC cell line HCT-116 and DLD-1 cells overexpressing Fascin-1 in vitro, while in vivo G2 inhibited invasion in zebrafish larvae xenografts [49].

Given the efficacy of G2 compound, improved analogues were developed. Notably NP-G2-011 and NP-G2-044 have been tested in vitro and in vivo with good efficacy, inhibiting migration and metastasis of breast cancer cells to the lungs, respectively [50]. As NP-G2-044 additionally exhibited good drug-like properties, it has moved to clinical trials. In a Phase 1A clinical trial (NCT03199586), NP-G2-044 was administered to patients with treatment-refractory solid tumor malignancies (https://clinicaltrials.gov/ct2/show/NCT03199586, accessed on 7 June 2023). In the trial that enrolled 23 patients, no serious adverse effects were observed from the administration of NP-G2-44, which was well tolerated, and gave preliminary signals of anti-tumor and anti-metastatic activity [51]. A phase 2A clinical trial with a particular focus on ovarian cancer is currently ongoing, at the phase of recruiting, which will test the efficacy of NP-G2-44 as a monotherapy or in combinations with anti-PD-L1 immune checkpoint inhibitors (NCT05023486 https://clinicaltrials.gov/ct2/show/NCT03199586, accessed on 7 June 2023).

Finally, two approved drugs have been identified as Fascin-1 inhibitors through repurposing. These include the antiviral drug raltegravir and the antidepressant imipramine. Both drugs inhibited in vitro migration and invasion of CRC cell lines HCT-116 and DLD-1 as well as invasion and metastasis in vivo, which was evaluated in the zebrafish model [52,53].

A summary of the above-described studies where Fascin-1 inhibitors were used is provided in Table 3 below.

## 2. Conclusions

Overall, there seems to be a strong and significant association between high Fascin-1 expression and a more invasive phenotype with poorer OS (Table 1) in many different cancer types. Moreover, both in vitro and in vivo experimentation using various cancer cell lines and tumor animal models have provided solid evidence in support of the notion that Fascin-1 promotes cancer cell metastasis and its inhibition can reverse metastasis-related properties (Table 2). In fact, the broad range of cancer types in which studies were performed with regard to Fascin-1, further strengthens the argument that targeting Fascin-1 is a promising strategy for inhibiting metastasis and cancer cell invasion across many different types of cancer.

From the studies analyzed in this review, there were only three studies that did not directly show an association between Fascin-1 expression and a metastatic phenotype or reduced OS. Specifically, in one study, although Fascin-1 was prominent in CRC samples and could be connected to unfavorable clinical outcome, the findings were not statistically significant [19]. The small number of samples (51 tissue samples) in this study may have accounted for this lack of significance in the findings. Similarly, no statistically significant differences were observed in a study involving prostate cancer samples from a tissue microarray even though Fascin-1 expression tended to be elevated more frequently in tumor cells proportional to high Gleason score [33]. In this study, 201 tissue samples were included and thus the number of samples is not small which leaves us with no apparent explanation for this difference compared to all other studies. A second study in prostate cancer patients also did not show significant difference but this involved blood and not tissue samples, and therefore, it is not fully comparable to the rest [34]. Thus, collectively in 10 different cancer types (Table 1), seven proved a robust association between Fascin-1 expression, poor survival and a metastatic phenotype, suggesting that Fascin-1 could be evaluated as a metastatic biomarker and could also prove to be a very potent anti-metastatic drug target.

In that regard, we also provide a summary of the approaches taken to pharmacologically inhibit Fascin-1 (Figure 2) in an attempt to block metastasis in vitro, in vivo and in clinical trials. Interestingly, the fact that Fascin-1 expression is barely non-existent in normal epithelial tissues [11] provides an additional advantage to its use as an anti-cancer target, as blocking Fascin-1 will not affect normal epithelial cells since they do not really express it. Therefore, more intensive research efforts are required for evaluating Fascin-1 inhibitors in various in vitro, in vivo or clinical settings. Fascin-1 is an extremely promising anti-metastatic therapeutic target that could potentially revolutionize anti-cancer therapy by increasing life expectancy of cancer patients while enhancing their life quality.

## Figures and Tables

**Table 3:** Studies on the effect of Fascin-1 inhibitors in cancer cell metastasis in vitro and/or in vivo.

Reference	Inhibitor	Cell Type	Methodology Used	Experimental Approach
[46]	Thiazole derivatives	MDA-MB-231 breast cancer cells	-Transwell migration assay -Transwell invasion assay	In vitro
			Angiogenesis: chick embryo ex ovo in chorioallantoic membrane (CAM) assay	Ex ovo
[47]	Tetrahydropyrimidine derivatives	4T1 breast cancer cells	-Wound healing assay	In vitro
[48]	Macroketone	4T1 breast cancer cells MDA-MB-231 breast cancer cells	-Wound healing assay -Transwell migration assay	In vitro
		4T1 breast cancer cells	6-thioguanine clonogenic assay, lung metastasis mice	In vivo
[11]	G2	MDA-MB-231, 4T1 breast cancer cells	-Transwell migration assay	In vitro
		4T1 breast cancer cells	6-thioguanine clonogenic assay, lung metastasis mice	In vivo
		MDA-MB-231 breast cancer cells	Whole animal imaging, metastasis monitoring in mice (GFP-luciferase cells)	
[45]	G2	HeyA8, Ovcar5 and Tyk-nu ovarian cancer cells, primary human cancer-associated fibroblasts (CAF) and primary human omental mesothelial cells (HPMC)	-Transwell migration assay -Oris™ cell migration assay -Wound healing assay	In vitro
		HeyA8 ovarian cancer cells	-Omental colonization assay -GFP/luciferase-labeled cells, tumor weight in mice -Metastasis monitoring	In vivo
[49]	G2	HCT-116 and DLD-1 CRC cells	-Wound healing assay -Transwell invasion assay	In vitro
			Zebrafish invasion model	In vivo
[50]	G2 NP-G2-011 NP-G2-036 NP-G2-044 NP-G2-050	Human MDA-MB-231 and Mouse	-Transwell migration assay	In vitro
		4T1 breast cancer cells	-Tumor metastasis in mice	In vivo
[51]	NP-G2-044	23 patients with treatment-refractory solid tumor malignancies		*Phase 1A clinical trial*
[52]	Raltegravir	HCT-116 and DLD-1 CRC cells	-Wound healing assay -Transwell invasion assay	In vitro
			-Zebrafish invasion and metastasis assay	In vivo
[53]	Imipramine	SW480, DLD-1 and HCT116 CRC cells	-Wound healing assay	In vitro
		HCT116 CRC cells	-Transwell invasion assay	
			-Myoma organotypic invasion model	
		DLD-1 and HCT116 CRC cells	-Zebrafish invasion and metastasis assay	In vivo

## Abbreviations

AFM	Atomic force microscopy
CRC	Colorectal cancer
ECM	Extracellular matrix
EMT	Epithelial to mesenchymal transition
ER	Estrogen receptor
FISH	Fluorescence in situ hybridization
HER-2	Human epidermal growth factor receptor-2
HIF	Hypoxia inducible factor
HSCCa	Hypopharyngeal squamous cell carcinoma
IC50	Half-maximal inhibitory concentration
IDC	Invasive ductal carcinoma
IGF	Insulin growth factor
IL-6	Interleukin 6
KPC	Kras(G12D) p53(R172H)Pdx1
LINC	Linker of nucleoskeleton and cytoskeleton
MAPK	Mitogen activated protein kinase
MMP-9	Matrix metalloproteinase-9
NSCLC	Non-small cell lung cancer
OS	Overall survival
HSCC	Hypopharyngeal squamous cell carcinoma
OV	Ovarian cancer
PanIN	Pancreatic intraepithelial neoplasia
PDAC	Pancreatic ductal adenocarcinoma
PR	Progesterone receptor
PSA	Prostate-specific antigen
RFS	Remission-free survival
SCID	Severe combined immunodeficient mice
SCLC	Small cell lung cancer
shRNA	Short-hairpin RNA
SNP	Single nucleotide polymorphisms
TGF-β	Transforming growth factor β
TNBC	Triple negative breast cancer
